# A comparative assessment of two different front-of-package nutrition label designs: A randomized experiment in Brazil

**DOI:** 10.1371/journal.pone.0265990

**Published:** 2022-04-06

**Authors:** Neha Khandpur, Laís Amaral Mais, Ana Paula Bortoletto Martins

**Affiliations:** 1 Faculty of Public Health (FSP), Department of Nutrition, Department of Nutrition, Center for Epidemiological Studies in Health and Nutrition (Nupens), University of São Paulo (USP), São Paulo, Brazil; 2 Department of Nutrition, Harvard, T. H. Chan School of Public Health, Boston, Massachusetts, United States of America; 3 Brazilian Institute for Consumer Defense (Idec), São Paulo, Brazil; The University of Hong Kong, HONG KONG

## Abstract

This study compares the effectiveness of different front-of-package label designs in a Brazilian sample (n = 1384). Eligible adults were randomized to one of two study arms and viewed images of snacks with either a triangular warning label (TL) or a rectangular ‘high in’ label with a magnifying glass (ML). They responded to a series of questions that captured label usefulness, understanding, and purchase intentions. Compared to participants in the ML arm, those in the TL arm agreed that the TL communicated important information [Mean (SD) - 5.47 (0.07) vs 4.49 (0.08), p-value <0.001], was a useful tool [Mean (SD) - 6.12 (0.06) vs 5.75 (0.07), p-value <0.001], and was easier to understand as measured subjectively [Mean (SD) - 4.96 (0.07) vs 4.44 (0.08), p-value <0.001]. However, both the TL and the ML performed similarly in communicating nutrient information as measured by the objective understanding of nutrient content [57.09% vs 54.65%, p-value 0.259]. The ML performed marginally better at improving purchase intentions [Mean (SD) - 2.57 (0.07) vs 2.79 (0.08), p-value <0.049]. The current study adds to the growing evidence base on the pathways through which FoP labels, particularly ‘high in’ labels, might influence consumer perceptions and behavior. It is also one of the first studies to provide evidence on the utility of the ML design for Brazil.

## Introduction

Front-of-package (FoP) nutrition labels have been recommended by international health organizations like the World Health Organization (WHO) and the Pan American Health Organization (PAHO), as a cost-effective policy to promote healthy food selection at points-of-purchase [1–3]. FoP nutrition labels come in many forms including icons, logos or symbols and are designed to feature prominently on the package and provide simplified nutrition information, complementing that of the nutrition facts panel and the list of ingredients [4,5]. Their salience and accessibility allows consumers to differentiate between products that are healthy from those that are less healthy, discouraging the purchase of the later. FoP labels also encourage food manufacturers to reformulate their products towards options that have a generally healthier nutrient profile [6].

Among the different models of FoP labels implemented globally, a relatively new paradigm of FoP labels, best represented by the warning label model implemented in Chile in 2016, stands out as being one of the more effective FoP labels for identifying nutrients of public health concern [7]. These warning labels alert the consumer to products with excess amounts of critical nutrients of public health concern like total sugars, saturated fat, and sodium [8]. Depending on the context, they may also be used to call attention to the presence of artificial sweeteners or the caloric content of the product like they do in Mexico [9]. Using the theoretical model proposed by Taille et al. as the basis of judging label effectiveness [10], warning labels have generally been shown to be more visually noticeable, better comprehended, more effective at decreasing perceptions of healthfulness of unhealthy products and more helpful at selecting the healthier of two products, compared to the multiple-traffic light model [11–13], with some exceptions [14,15]. In experimental studies, warning labels have shown to decrease consumers’ purchases of less healthy products compared to other labels and significantly reduce the calorie and sugar content of purchased products compared to no label [11,16].

Sales data and information from nutrition labels of products from contexts that have implemented warning labels, provide further support for these experimental results. Results from the evaluation of the Chilean Law of Food Labeling and Advertising that includes restrictions on marketing to children, banning in-school sales, and the mandatory implementation of warning labels on packaged ultra-processed food and beverage products (UPP) high in calories, saturated fat, sodium, and sugar found a 23.7% drop in sugary drink purchases within the first year of the implementation, larger than those observed after the implementation of taxes [17]. An evaluation comparing the pre- and post-implementation period found an improvement in the nutrition profile of products, with a 7% reduction in the proportion of products containing an excess of energy, sugars, saturated fats, or sodium [18].

A number of factors contribute to the relative success of FoP label policies, including the nutritional profile model used, the number and type of products targeted, and the form of policy implementation [8]. The design elements of the FoP warning label, such as the colors used, the text displayed and the shape of the icon, may also contribute to its success [19]. Countries around the world have introduced a range of different FoP designs. For instance, the warning labels implemented in Chile, Peru, Mexico, and Uruguay are all in black–and–white. Models proposed in Canada also use the same contrasting colors [20]. Peru and Chile have warning labels that display the text ‘High in [X nutrient]’, as does the proposed model in Canada, while the models in Uruguay and Mexico read ‘Excess [X nutrient]’ [20]. Peru, Chile, Uruguay, and Mexico use octagons as their symbol–an internationally recognized symbol for ‘stop’ [21]. A similar label design proposed in Canada on the other hand, uses a magnifying glass [22]. A key difference between the magnifying glass FoP label and the octagon labels lies in the presentation of nutrients of excess—a separate octagon is presented for every nutrient that exceeds established thresholds, while the magnifying glass model identifies different nutrients in excess on a separate line, one below the other.

In Brazil, several models of warning labels have been put forward by different entities for consideration by the National Health Surveillance Agency (*Agência Nacional de Vigilância Sanitária*—Anvisa). Among them was the red circular icon with the text ‘High level of [X nutrient]’, a black-and-white octagon symbol with ‘Contains a lot of [X nutrient]’, and a black-and-white triangle with the text ‘High in [X nutrient]’ [23]. Along with the octagon, the triangle model has been studied in some detail and found to be a robust choice for the Brazilian population [24,25]. The magnifying glass model with the text ‘High in [X nutrient], put forth by Anvisa in 2020 as the FoP label for Brazil [26], has not been researched as extensively [27]. This study was conducted to provide more evidence on the relative utility of the magnifying glass model. The aim of the study was to compare the effectiveness of two different FoP label designs, the magnifying glass model and the triangle model, in improving understanding, perceptions, and purchase intentions of Brazilian consumers. It was hypothesized that the triangle model and its different design elements would perform better than the more neutral magnifying glass model. The triangular symbol, much like the octagon, is inherently informative independent of the signal phrase used and conveys ‘alert’ or ‘caution’ [28]. The presence of additional triangles for every nutrient in excess may play a role in reinforcing product avoidance. The magnifying glass was hypothesized to be a less visually alerting symbol.

## Methods

### Study design

**Fig 1:**
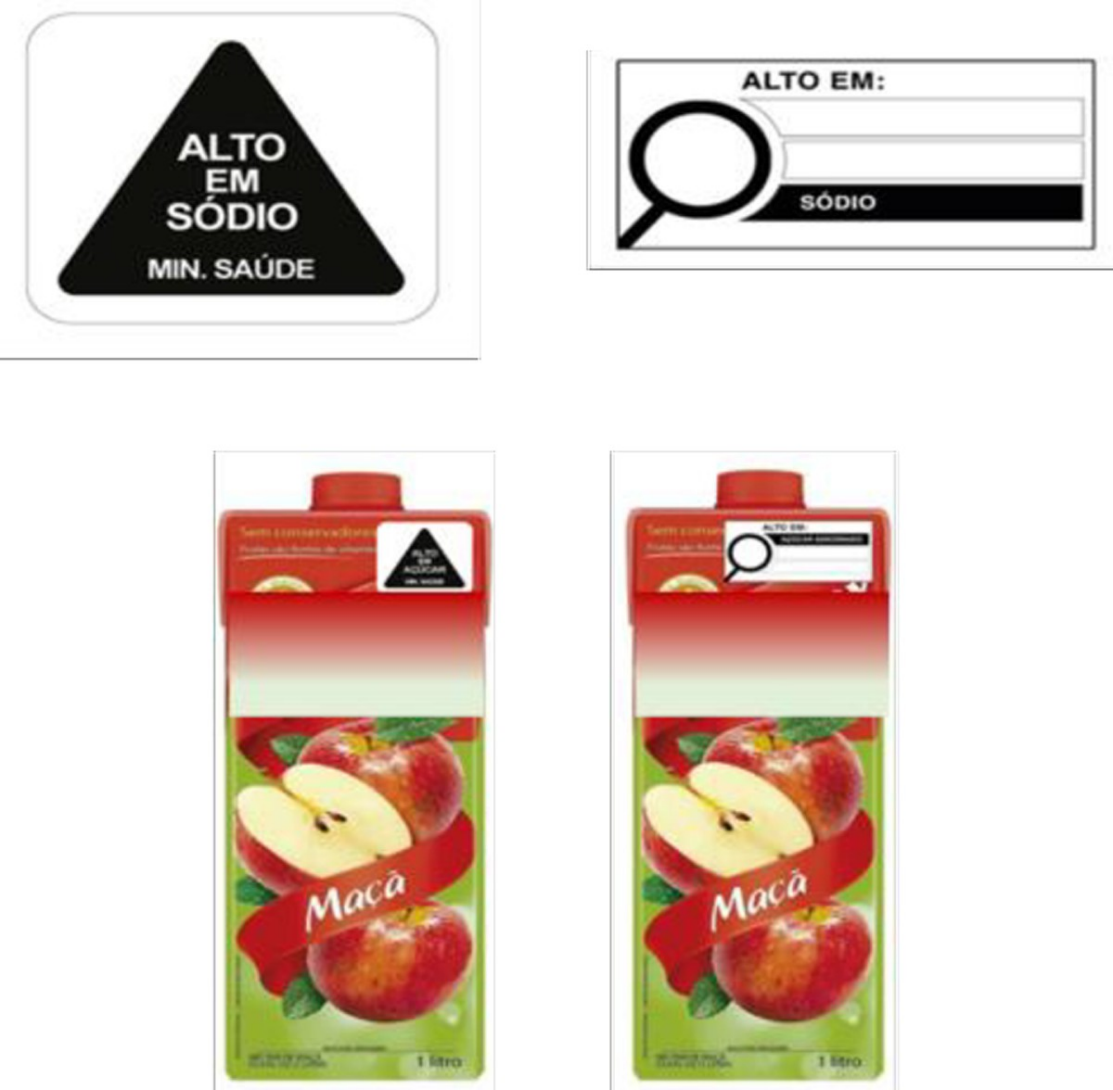
The triangular label and the magnifying glass label (top), examples of images used in the study (bottom).

A between-person randomized experiment was used to achieve the study objectives. In this design, all participants were exposed to one of two study conditions that presented the two different FoP label designs–a triangular, black-and-white warning label (henceforth called the triangular label, TL), or a rectangular, black-and-white ‘high in’ label with a magnifying glass (henceforth called the magnifying glass label, ML). The TL was developed by researchers in information design at the Federal University of Paraná (*Universidade Federal do Paraná*—UFPR), Brazil. The ML was developed by Anvisa in 2019 as part of the regulatory process for revising the nutrition labelling norms in Brazil (see Fig 1). This design is similar to the options for FoP labels under consideration by the Canadian government [22]. For this study, these labels were Photoshopped to appear on images of actual products available in Brazilian supermarkets and were presented to participants as part of a survey. Eligible individuals who verbally consented to participate in the survey saw identical product images but with different FoP labels depending on the study conditions they were randomized to and responded to an identical set of questions that appeared in the same sequence in both study conditions. The PAHO nutrient profile model was used to identify nutrients in excess for both label designs. The study procedures were approved by the University of São Paulo IRB. Obtaining verbal consent was deemed sufficient for this opinion-based survey.

### Participant recruitment

A Brazilian survey research firm was contracted to recruit participants from 101 cities representing the five macro-regions of the country, from points in the city with a large pedestrian population (outside supermarkets, metro stations, bus stops etc). Adults, between 18–55 years of age, across both sexes were recruited to make the sample more representative of the general Brazilian population with regards to sex and age. Individuals who were associated with the food industry, or those who currently worked in market research, or for an advertising agency, or for the media were not eligible to participate. In total 1,384 eligible adults consented and were included in the study sample. No reimbursement was provided for study participation. The study was administered in Portuguese, in October of 2019.

### Study procedures

A survey was developed for the purposes of data collection and was administered by trained interviewers, in-person. Immediately after receiving verbal consent, eligible participants were randomly assigned to one of the two study conditions, based on a randomly generated sequence, pre-loaded into the handheld tablet that the interviewers used to administer the study. Interviewers read out the survey questions from the tablet, showed participants product images associated with the study condition that they were assigned to on the screen, and read out response options where appropriate. Participant responses were recorded on the tablet by the interviewer after every question.

The first half of the survey collected information on participant demographics. The second half of the survey recorded participants’ responses on 10 questions. For five of these questions, participants were shown just an image of the label (either the TL or the ML depending on their random assignment) and asked about: (i) the importance of the information it communicated; (ii) the usefulness of the label in helping people make healthier food choices; (iii) the ease of understanding of the label for the general Brazilian population; (iv) how worried they would be if their child or a child in their family were to consume foods and beverages that displayed the label; and (v) how their purchases would be affected if a food or drink they frequently bought, exhibited this label. For all five questions, responses were recorded on a seven-point Likert scale. Participants were also shown two images, one at a time–one of an apple flavoured beverage with a label indicating excess of sugar and another of a packet of chips with a label indicating excess of sodium and saturated fat. For each product, they were asked to indicate what nutrient they thought was in excess in the product, if any. Response options provided were: excess of sodium; excess of saturated fat; excess of sugar; excess of saturated fat and sugar; excess of saturated fat and sodium; do not know. The other questions had participants see a pair of images of products from the same food category–two different brands of orange juice, two different brands of flavoured yogurt, and two different brands of breakfast cereal. For each product pair, participants were asked to identify the healthier option. Finally, an image of both the TL and the ML was shown side by side, and participants were asked to indicate the label they preferred to see on products that had excess amounts of sodium. All study participants saw an image of both labels for this question, irrespective of the study condition that they were assigned to and were asked to justify their choice of label in the only open text response option of the survey.

### Study outcomes

The following subjective (those that captured participant opinion and had no ‘correct’ response) and objective (questions with a single correct response) outcomes were studied: (1) Information communicated by the FoP label–this was a subjective outcome, with response options ranging from 1 (information not at all important) to 7 (information very important); (2) Usefulness of label—this outcome was measured using a subjective indicator, with response options ranging from 1 (label not at all useful) to 7 (label very useful); (3) Understanding of nutrient content–this outcome was captured using both an objective question and a subjective question. Responses for correctly identifying nutrients in excess in the images were given a score of 1 (0 for incorrect), added up, and converted into a mean percentage score (0–100), for the objective indicator. The subjective indicator took on a value between 1 (label very difficult to understand) to 7 (label very easy to understand); (4) Understanding of product healthfulness–this was an objective outcome, created by summing the correct responses to the product pair comparison questions and converting the responses into a mean percentage score (0–100); (5) Concern for child consumption–this subjective outcome had a value between 1 (not concerned) to 7 (very concerned); and (6) Purchase intentions–this was also a subjective outcome with a value between 1 (continue to buy) to 7 (stop buying).

### Analysis

Descriptive statistics were used to profile the study sample. T-tests and one-way ANOVAs were used to assess the statistical significance in the differences in continuous mean responses between study conditions. Chi-square tests were used for categorical variables. Differences between study conditions by sex, age group, and education attainment were also explored. Survey weights were used where appropriate and study hypotheses were tested using a two-tailed level of significance with p≤0.05. All analyses were conducted in Stata v.14 (StataCorp LLC, College Station, TX, USA). Open text responses were analysed inductively using thematic analysis in Microsoft Excel (Microsoft 365, academic license).

## Results

### Study demographics

Demographics of the study population can be found in Table 1. The total sample of 1,384 adults, of which the majority were women (51.4%), had a mean age of 35.4 years (±10.8). The sample was well-educated with over 80% having completed either secondary or tertiary level of education, and a majority of the sample was employed; however, 70% of the sample reported low income. A total of 694 were randomized to the ML while 690 participants were randomized to the TL. There were no differences by study condition for participants’ sex, age, family income, education, occupation, and their geographic region. There was, however, a significant difference in whether the participants had children. This variable was controlled for in all subsequent analysis, but it did not change the direction or the magnitude of the results (data not shown).

**Table 1:** Study demographics.

Indicators	Total sample n = 1,384	Study arms		Comparing study arms
		Magnifying glass label n = 694	Triangular label n = 690	p-value
Age, mean yrs (SD)	35.38 (10.83)	35.79 (10.65)	34.97 (10.99)	0.15
Sex, %				
Female	51.37	52.45	50.29	0.42
Male	48.63	47.55	49.71	
Education, %				
Primary or less	17.27	16.86	17.68	0.84
Secondary	59.18	59.94	58.41	
Tertiary	23.55	23.20	23.91	
Income, %				
Low	70.16	69.02	71.30	0.23
Medium	26.73	28.39	25.07	
High	3.11	2.59	3.62	
Occupation, %				
Employed	80.13	79.11	81.16	0.50
Unemployed	18.42	19.16	17.68	
Retired	1.45	1.73	1.16	
Have children, %				
No	36.92	34.29	39.57	**0.04**
Yes	63.08	65.71	60.43	
Geographic region, %				
Midwest	8.67	8.65	8.70	1.00
Northeast	25.94	25.94	25.94	
North	8.53	8.65	8.41	
Southeast	42.34	42.36	42.32	
South	14.52	14.41	14.64	

SD: Standard deviation.

Values in bold are statistically significant (p≤0.05).

### Differences in study outcomes between label designs

Information communicated by the FoP label: Participants in the TL arm scored significantly higher than those in the ML arm for this outcome, indicating stronger agreement with the statement that the label communicated important information [Mean (SD) on a scale of 1–7 = 5.47 (0.07) vs 4.49 (0.08), p-value <0.001].

Usefulness of label: Participants in the TL arm perceived the label to be significantly more useful than participants in the ML arm [Mean (SD) on a scale of 1–7 = 6.12 (0.06) vs 5.75 (0.07), p-value <0.001].

Understanding of nutrient content: There were no significant differences in the objective understanding of nutrient content between study arms. Participants’ ability to identify excess nutrients in the products they were shown was similar for both the TL and the ML [57.09% vs 54.65%, p-value 0.259]. However, when asked how easy would it be for the general Brazilian population to understand this label, participants in the TL arm rated their label higher than those in the ML arm [Mean (SD) on a scale of 1–7 = 4.96 (0.07) vs 4.44 (0.08), p-value <0.001].

Understanding of product healthfulness: In the product comparison task, participants’ ability to identify the healthier of the two products was significantly higher in the TL arm compared to those in the ML arm as indicated by their higher scores on this outcome [79.47% vs 67.91%, p-value <0.001].

Concern for child consumption: Participants in the TL arm reported that they would be significantly more worried if the children in their family were to consume products carrying this label than participants in the ML arm [Mean (SD) on a scale of 1–7 = 6.13 (0.06) vs 5.89 (0.07), p-value <0.012].

Purchase intentions: Participants in the TL arm seemed marginally more likely to continue to purchase a frequently bought food or beverage product, even if it carried a TL compared to participants in the ML arm [Mean (SD) on a scale of 1–7 = 2.57 (0.07) vs Mean (SD) on a scale of 1–7 = 2.79 (0.08), p-value <0.049]. More details can be found in Table 2.

**Table 2:** Study outcomes across arms.

Outcomes	Magnifying glass label n = 694	Triangular label n = 690	F-statistic (p-value)
	Means (SE)			
**Importance of information communicated** 1 not important– 7 very important	4.49 (0.08)	5.47 (0.07)	78.12 (<0.001)
**Usefulness of label** 1 not useful– 7 very useful	5.75 (0.07)	6.12 (0.06)	15.74 (<0.001)
**Understanding of nutrient content**		
Objective score 0–100	54.65 (1.51)	57.09 (1.52)	1.28 (0.25)
1 not easy to understand– 7 very easy to understand	4.44 (0.08)	4.96 (0.07)	21.73 (<0.001)
**Understanding of product healthfulness** 0–100	67.91 (1.13)	79.47 (1.01)	58.01 (<0.001)
**Concern for child consumption** 1 not concerned– 7 very concerned	5.89 (0.07)	6.13 (0.06)	6.29 (0.01)
**Purchase intentions** 1 continue to buy– 7 stop buying	2.79 (0.08)	2.57 (0.07)	3.88 (0.04)

SE: Standard errors.

In exploratory analysis, participants with the highest educational attainment scored the highest in the objective understanding of nutrient content and product healthfulness and were most concerned about the intake of products containing labels by children, compared to those with lower educational attainment. There were no significant differences in purchase intentions by education attainment. Women and participants in the 18-24-year age range followed a similar pattern when compared to men and participants in the older age groups, respectively. Women and the youngest participants reported significantly higher scores on label usefulness, and on objective understanding of nutrient content. Women also reported greater concern for children consuming labelled products but were less likely to change purchasing habits of these products. In contrast, 18-24-year-old participants reported higher intentions to stop buying labelled products compared to their older counterparts. They also scored significantly higher on their understanding of product healthfulness (data not shown).

Choice of label: When shown pictures of both labels, almost 73% of the participants from the TL arm selected the same label design over the ML design while nearly 79% of the participants from the ML arm switched preferences and chose the TL. The percentage of participants in the ML arm who said they would choose the TL as their preference for a FoP label on products that had excess of sodium was significantly high [78.95% vs 72.49%, F-statistic = 7.66, p-value = 0.005].

Participants cited various reasons for their choice of label. These reasons were coded into the following response categories: “conspicuous, salient, clearly identifiable”; “easier to understand”; “more informative”; “wording was impactful” and “symbol was impactful”. The presence of the Ministry of Health logo, present only on the TL, added credibility to the information presented on the label and was another reason why this design was chosen (see Fig 1). In some instances, respondents just “liked the label better”, without being specific about why they thought so.

Among participants in the ML arm, those who continued to support the ML design did so because they thought ‘*the symbol was different and new and interesting*’; ‘*the magnifying glass gives the idea of warning to search more*’; and ‘*because of the amount of information*. *The other label [TL] is more attractive*, *but it gives less information*’. Respondents who had used the ML to respond to survey questions but chose the TL design did so because they *‘…found the second [ML] vague and confusing*. *It would go unnoticed*. *The first [TL] would draw more attention*’. The TL would also be better noticed by *‘…those who have weak vision’*. Others chose the TL because ‘*the [ML] seems just common information and the [TL] seems more like an alert*’. According to one participant, ‘*I found this simpler to understand*, *draws attention while the other [ML] has very small writing*’.

Participants in the TL arm that chose the TL design over the ML thought that the TL was a ‘*better recognized symbol*. *The other [ML] looks like a business card*’ and ‘… *was practically blank*’. The symbol and the wording on the TL ‘*make it possible for me to read*. *The other [ML]*, *I struggle to read*. *People who cannot see well or do not know how to read at all will have a better idea of … the product [with the TL]*.’ Respondents in the TL arm that chose the ML ‘*found it more interesting’*. The design ‘*attracts curiosity*’ and can ‘*contain more information*’. One participant reported choosing the ML because ‘*the image looks less aggressive*’ than the TL. A more detailed breakdown of the codes across study arms is presented in Table 3.

**Table 3:** Reasons for choice of front-of-package label by study arm.

Reasons	Magnifying glass label n = 694		Triangular label n = 690	
	Choice of ML	Choice of TL	Choice of ML	Choice of TL
	n (%)				
Conspicuous, salient, clearly identifiable	24 (3.45)	186 (26.76)	35 (5.07)	199 (28.84)
Easier to understand	8 (1.15)	21 (3.02)	10 (1.44)	37 (5.36)
I like it better	4 (0.57)	4 (0.57)	9 (1.30)	2 (0.28)
More informative	73 (10.50)	125 (17.98)	93 (13.47)	103 (14.92)
Wording is impactful	1 (0.14)	25 (3.59)	2 (0.28)	30 (4.34)
Ministry of Health	0 (0)	59 (8.48)	0 (0)	31 (4.49)
Symbol is impactful	19 (2.73)	108 (15.53)	21 (3.04)	92 (13.33)
Other	2 (0.28)	2 (0.28)	5 (0.72)	1 (0.14)
Do not know	14 (2.01)	20 (2.87)	13 (1.88)	7 (1.01)
**Total**	**145 (21.05)**	**550 (78.95)**	**188 (27.51)**	**502 (72.49)**

## Discussion

The aim of this study was to demonstrate the relative effectiveness of two FoP nutrition labels that indicated an excess of nutrients—the ML model and the TL model—in improving consumer understanding, perceptions, and purchase intentions. In this sample of Brazilian adults the TL performed better on most study outcomes. However, both the TL and the ML performed similarly in communicating nutrient information as measured by the indicator on objective understanding of nutrient content and the ML performed marginally better at improving purchase intentions. Qualitative evidence indicated that the TL was the overall preferred label design for participants in both study conditions.

Participants in this study strongly agreed that in comparison to the ML, the TL was more effective at communicating important information, was a useful tool, and was easier to understand as measured subjectively. Published literature largely supports warning labels as being equally useful or positively perceived by study participants, compared to other labels like the Nutri-Score, the multiple traffic light label or the Health Star Rating [15,29]. In some instances, the TL has out-performed other labels like the multiple traffic-light label in terms of perceived label utility [12]. In the current study, the TL was the preferred label among participants, even among those who saw the ML for all their tasks—a larger portion of participants in the ML study arm indicated their preference for the TL when given a choice. Quantitative evidence from the Canadian study supports a similar conclusion to the one seen in the current study–a red warning label was perceived as being more effective than the magnifying glass for informing consumers that a product had an excess of saturated fat or sugar [30]. However, more recent work from a Brazilian sample showed comparatively lower consumer perceptions of the TL compared to the ML with regards to it informing or enabling decision making, based on qualitative responses to an open-ended question [25].

In the current study, objectively measured understanding that captured participants’ ability to correctly identify nutrients in excess, found no differences between the TL or the ML. The similarity in the performance between the ML and the TL in helping to identify nutrient content above nutritional recommendations has been shown in two other studies [25,31]. A Canadian study showed the ML as being superior to a no-label control in helping participants identify foods high in saturated fat, sugars, and/or sodium [27]. However, a different study conducted with a different Canadian sample found that the ML was no different from a no-label control in helping participants identify the product with an excess of saturated fat or sugar [30]. A red octagon performed the best of 5 different models. A similar result was demonstrated in a sample from Jamaica, where the chances of correctly identifying a product containing excessive amounts of critical nutrients were nine times higher with a black octagonal warning compared to a Facts Up Front model (that stated nutrient information in grams per portion with no other interpretative element) and only 5.8 times higher with a ML [32].

In the current study the TL was more effective than the ML in getting participants to identify the healthier of the two products. There seems to be some consensus in the published literature with respect to the ML on objectively measured comparative tasks. The ML was found to be no different from a Facts Up Front control in correctly identifying the least harmful products in the Jamaican study, while an octagon warning label was at least twice as good [32]. The ML was also inferior to the TL in improving consumer ability to identify the most healthful product in the study by Deliza et al. [25].

Participants in the current study also reported being significantly more worried if the children in their families consumed products carrying this label compared to the ML. However, contrary to expectations, they also seemed less likely reduce purchases and to give up buying products if it carried the TL. These results run contrary to the results from the Jamaican sample in which, compared to the control, the chances of deciding to purchase the least harmful option or none of the options were higher for the octagonal warnings than they were for the ML [32]. The Jamaican study did not include a TL but an octagon warning label which the TL closely resembles in terms of performance. The ML was shown to be equivalent to the TL in improving purchase intentions compared to a no-label control condition, in a different sample of Brazilian adults [31]. These varying results suggest that more work is needed to uncover if the ML could potentially improve consumer purchase intentions.

The results seen may be attributed to certain design elements of FoP labels. Triangles and octagons are familiar in road signs and communicate ‘alert’, ‘caution’ or ‘warning’. The magnifying glass by comparison is less intuitively alerting and may have underperformed on some study indicators for this reason. Despite this, the ML with the magnifying symbol, did do marginally better at encouraging consumers to reduce their purchase intentions. The black-and-white contrasting colours, the position on the package, the white background to offset the FoP label from the rest of the product packaging, and the large size are other design elements that have shown to be important at capturing consumer attention [19] and were features that were common to both labels in this study. These aspects may have helped the ML perform similarly to the TL. Another aspect to consider is the non-random sequence of the questions–the question on purchase intentions was asked at the very end, allowing participants in both groups to think more deeply about their responses. This may be another reason for the similarity in responses in both study arms.

The limitations of this work need to be considered. The study only included a limited range of products presented as two-dimensional images on tablets. Future studies may consider including a greater diversity of products to ascertain that the results seen here are applicable across a wider product portfolio in real-world settings, with recruitment across different contexts. The survey was interviewer administered which may have introduced some level of social desirability bias in the responses, although there was no evidence to suggest that this differentially impacted the results. The ML design tested was the version endorsed by Anvisa in 2019 [23], and not the slightly updated version they presented as part of their nutrition labelling regulation in 2020 [26]. The latest version of the ML has fewer blank spaces, with the image of the magnifying glass and the words ‘High in’ in a white box, separate from the nutrients in excess in the product. The nutrients in excess are displayed in separate black rectangular boxes, alongside or below the magnifying glass. While likely to be similar, this study may need to be replicated to confirm results and have them be applicable to the 2020 ML design. The study benefitted from random assignment to study conditions and a large sample recruited from different regions in Brazil, ensuring geographic diversity, with representation from varied income groups and participants with different education attainments.

The weight of the evidence suggests that the 2020 version of the ML is unlikely to be the strongest FoP label for the Brazilian context. However, it has several design elements that are similar to the 2019 version of the ML tested in this study. Extrapolating the results from this study, the 2020 ML may support improved consumer understanding and guide consumers toward healthier food purchases, provided the label complies with minimum size requirements, is displayed in a standardized manner on appropriate food packages, and is accompanied by a strong nutrient profiling system. Future studies will be needed to confirm this hypothesis, and to monitor the implementation and evaluate the impact of the Brazilian labelling policy.

## Conclusions

The current study adds to the growing evidence base on the pathways through which FoP nutrition labels, particularly ‘high in’ labels, might influence consumer perceptions and behavior. It is also one of the first studies to provide evidence on the utility of the ML design for Brazil. The ML was shown to be marginally more effective at decreasing purchase intentions than the TL in this sample of participants and was equal to the TL in helping participants correctly identify nutrients in excess. The TL did better than the ML at eliciting a positive assessment from the participants and in helping them identify the healthier of the two products. Going forward, experimental studies that capture the behavioral response of consumers like changes in product purchases in real-world settings, and dietary intakes and changes to nutrient compositions of food products, will be crucial to upholding and improving Anvisa’s final FoP label policy.

## Supporting information

S1 Table Original study questionnaire in Portuguese. (DOCX)

S1 Dataset (XLSX)
