# Supplementary material for: A comparative assessment of two different front-of-package nutrition label designs: A randomized experiment in Brazil
Source: PLoS One. 2022 Apr 6;17(4):e0265990. doi: 10.1371/journal.pone.0265990 (PMC8985949; doi:10.1371/journal.pone.0265990)
Supplement: S1 Table — (DOCX) [file pone.0265990.s001.docx]

Original questionnaire in Portuguese

| A Agência Nacional de Vigilância Sanitária, do Ministério da Saúde, deve determinar nos próximos meses que rótulos de alimentos e bebidas que contenham grande quantidade de sódio, açúcar ou gordura saturada devem ter um símbolo, com destaque, na parte da frente das embalagens.  Pensando nisso, eu gostaria que você respondesse algumas perguntas. | | |
| --- | --- | --- |
| No. | Pergunta | Respostas |
| 1 | Pensando numa escala de 1 a 7 em que 1 é nenhuma informação e 7 muita informação, o quanto de informação esse símbolo traz para você? | \| 1 \| 2 \| 3 \| 4 \| 5 \| 6 \| 7 \| 99.Não sabe \| \| --- \| --- \| --- \| --- \| --- \| --- \| --- \| --- \| |
| 2 | Na sua opinião, qual das respostas deste cartão é mais apropriada para este produto:  [1 Suco] | 1 O produto da imagem tem excesso de sódio  2 O produto da imagem tem excesso de gordura  3 O produto da imagem tem excesso de açúcar  4 O produto da imagem tem excesso de gordura e sódio  5 O produto da imagem tem excesso de gordura e açúcar  99. Não sabe |
| 3 | Na sua opinião, qual das respostas deste cartão é mais apropriada para este produto:  [1 Salgado] | 1 O produto da imagem tem excesso de sódio  2 O produto da imagem tem excesso de gordura  3 O produto da imagem tem excesso de açúcar  4 O produto da imagem tem excesso de gordura e sódio  5 O produto da imagem tem excesso de gordura e açúcar  99. Não sabe |
| 4 | Pensando numa escala em que 1 significa muito difícil de compreender e 7 muito fácil de compreender, o quanto você acha que a população brasileira irá compreender este símbolo? | \| 1 \| 2 \| 3 \| 4 \| 5 \| 6 \| 7 \| 99.Não sabe \| \| --- \| --- \| --- \| --- \| --- \| --- \| --- \| --- \| |
| 5 | Na sua opinião, qual destes produtos é o **MENOS SAUDÁVEL**?  [2 Sucos] |  |
| 6 | Na sua opinião, qual destes produtos é o **MAIS SAUDÁVEL**?  [2 Iogurtes] |  |
| 7 | Na sua opinião, qual destes produtos é o **MENOS SAUDÁVEL**?  [2 Cereais do café da manhã] |  |
|  | Agora eu gostaria que você avaliasse esse símbolo, que poderia estar no rótulo de alguns alimentos para indicar que possuem muito açúcar, gordura saturada ou sódio  [Imagen do FoP] | |
| 8 | Pensando numa escala em que 1 é nada útil e 7 é muito útil, como você avalia a utilidade desse símbolo para ajudar as pessoas a fazer escolhas alimentares saudáveis? | \| 1 \| 2 \| 3 \| 4 \| 5 \| 6 \| 7 \| 99.Não sabe \| \| --- \| --- \| --- \| --- \| --- \| --- \| --- \| --- \| |
| 9 | Pensando numa escala em que 1 significa nenhuma preocupação e 7 muita preocupação, o quanto você se preocuparia se seus filhos ou crianças de sua família consumisse alimentos e bebidas que tenham esse símbolo? | \| 1 \| 2 \| 3 \| 4 \| 5 \| 6 \| 7 \| 99.Não sabe \| \| --- \| --- \| --- \| --- \| --- \| --- \| --- \| --- \| |
| 10 | Pensando numa escala em que 1 com certeza continuaria comprando e 7 com certeza deixaria de comprar, o que você faria se uma bebida ou comida que você compra frequentemente exibisse esse símbolo? | \| 1 \| 2 \| 3 \| 4 \| 5 \| 6 \| 7 \| 99.Não sabe \| \| --- \| --- \| --- \| --- \| --- \| --- \| --- \| --- \| |
| 11 | Se você pudesse escolher um símbolo para destacar em rótulos de produtos que tenham quantidades elevadas de sódio, qual desses você escolheria?  [Imagens dos FoPs] |  |
| 12 | Por quais motivos você escolheu esse símbolo?  **(RESPOSTA ESPONTÂNEA E MÚLTIPLA)** |  |

Translated version of the questionnaire in English

| The National Health Surveillance Agency of the Brazilian Ministry of Health will determine a new label that food and beverages with large amounts of sodium, sugar or saturated fat must prominently display, on the front of the package.  With that in mind, I would like you to answer a few questions. | | |
| --- | --- | --- |
| Sl.no. | Question | Response options |
| 1 | On a scale from 1 to 7 where 1 is no information and 7 is a lot of information, how much information does this symbol convey? | \| 1 \| 2 \| 3 \| 4 \| 5 \| 6 \| 7 \| 99.Don’t know \| \| --- \| --- \| --- \| --- \| --- \| --- \| --- \| --- \| |
| 2 | In your opinion, which of these responses is most appropriate for the following product:  [1 Juice] | 1 The product in the image has excess sodium  2 The product in the image has excess fat  3 The product in the image has excess sugar  4 The product in the image has excess fat and sodium  5 The product in the image has excess fat and sugar  99. Don't know |
| 3 | In your opinion, which of these responses is most appropriate for the following product:  [1 Packet of chips] | 1 The product in the image has excess sodium  2 The product in the image has excess fat  3 The product in the image has excess sugar  4 The product in the image has excess fat and sodium  5 The product in the image has excess fat and sugar  99. Don't know |
| 4 | On a scale from 1 to 7 where 1 means very difficult to understand and 7 very easy to understand, how easy is it for the Brazilian population to understand this symbol? | \| 1 \| 2 \| 3 \| 4 \| 5 \| 6 \| 7 \| 99.Don’t know \| \| --- \| --- \| --- \| --- \| --- \| --- \| --- \| --- \| |
| 5 | In your opinion, which of these products is the LESS HEALTHY?  [2 Juices] | Select image |
| 6 | In your opinion, which of these products is the HEALTHIER?  [2 Yogurts] | Select image |
| 7 | In your opinion, which of these products is the LESS HEALTHY?  [2 Breakfast cereals] | Select image |
|  | Now I would like you to review this symbol, which would appear on food packages to indicate that they have a lot of sugar, saturated fat or sodium.  [Image of FoP that participants were randomized to] | |
| 8 | On a scale from 1 to 7 where 1 is not at all useful and 7 is very useful, how would you rate the usefulness of this symbol in helping people make healthy food choices? | \| 1 \| 2 \| 3 \| 4 \| 5 \| 6 \| 7 \| 99.Don’t know \| \| --- \| --- \| --- \| --- \| --- \| --- \| --- \| --- \| |
| 9 | On a scale from 1 to 7 where 1 is not worried and 7 is very worried, how worried would you be if your children or children in your family consumed foods and drinks that have this symbol? | \| 1 \| 2 \| 3 \| 4 \| 5 \| 6 \| 7 \| 99.Don’t know \| \| --- \| --- \| --- \| --- \| --- \| --- \| --- \| --- \| |
| 10 | On a scale from 1 to 7 where 1 is would definitely keep buying and 7 is would definitely not buy, what would you do if a drink or food you buy frequently displayed this symbol? | \| 1 \| 2 \| 3 \| 4 \| 5 \| 6 \| 7 \| 99.Don’t know \| \| --- \| --- \| --- \| --- \| --- \| --- \| --- \| --- \| |
| 11 | If you could pick a symbol to stand out on product labels that have high amounts of sodium, which one would you choose?  [Images of both FoP labels] | Select image |
| 12 | What were your reasons to choose this symbol? | Open text |
